# Interference of dissolved organic matter and its constituents on the accurate determination of hydrogen peroxide in water

**DOI:** 10.1038/s41598-021-01016-9

**Published:** 2021-11-19

**Authors:** Jianbiao Peng, Ya Zhang, Jianhua Li, Xinan Wu, Mengjie Wang, Zhimin Gong, Shixiang Gao

**Affiliations:** 1 Nanjing Institute of Environmental Sciences, Ministry of Ecology and Environment of China, Nanjing, 210042 People’s Republic of China; 2 Key Laboratory for Yellow River and Huai River Water Environmental and Pollution Control, Ministry of Education, Henan Key Laboratory for Environmental Pollution Control, School of Environment, Henan Normal University, Xinxiang, 453007 People’s Republic of China; 3 Jiangsu Key Laboratory of Chemical Pollution Control and Resources Reuse, School of Environmental and Biological Engineering, Nanjing University of Science and Technology, Nanjing, 210094 People’s Republic of China; 4 State Key Laboratory of Pollution Control and Resource Reuse, School of the Environment, Nanjing University, Nanjing, 210023 People’s Republic of China

**Keywords:** Analytical chemistry, Environmental sciences

## Abstract

Hydrogen peroxide (H_2_O_2_) is ubiquitous in natural waters, and plays an important role in both biological and chemical processes. This study investigated the influence of dissolved organic matter (DOM) and its substituents on the accurate measurement of H_2_O_2_ by peroxidase-mediated depletion of scopoletin fluorescence method which is one of the most widely used methods for the determination of low concentration H_2_O_2_ in water. Six DOM and its 24 substituents interfered the determination of H_2_O_2_ at environmentally relevant concentration of 200 nM with different levels except 2,6-dimethoxy-1,4-benzoquinone and benzoic acid, which may be associated with origin and types of DOM, and numbers and position of active functional groups in DOM constituents. Each substance concentration and the corresponding decreasing ratio to the measured H_2_O_2_ concentration was fitted well to the linear model (R^2^ > 0.9), and the obtained interfering ratios (*k,* (mgC L^−1^)^−1^), expressing the degree of DOM or its substituents per unit concentration to the measurement of H_2_O_2_, were approximate for DOM, but the order of magnitude of *k* values of DOM constituents took on a large span from 10^–3^ to 10^–7^. When DOM levels exceed 0.1 mgC L^−1^ or its substituent concentration is at nM level (low to 20 nM), the H_2_O_2_ content will be underestimated substantially. A quantitative structure–activity relationship model with remarkable stability and strong predictability for the *k* of DOM substituents to H_2_O_2_ measurement was established, and the *k* was related to the electron transfer capacity, hydrophobicity and stability of these compounds.

## Introduction

As a relatively stable reactive oxygen species (ROS) with a long half-life (several to 100 h)^1^, hydrogen peroxide (H_2_O_2_) is ubiquitous in natural waters such as freshwater, rainwater, and seawater with a steady-state concentration ranging from nM to μM^2,3^. The source of H_2_O_2_ include the interactions of sunlight and light-absorbing substances (e.g., chromophoric dissolved organic matter (CDOM)), redox cycling of metals and biological processes, and atmospheric deposition^4–10^. The high redox-active transient plays an important role in the environmental fate of organic compounds and geochemical cycling of trace elements (e.g., iron, copper, chromium, manganese)^11–13^, thereby indirectly affecting their biological availability and/or toxicity to organisms^14^. Also, H_2_O_2_ can pose deleterious effects on biological systems directly. For instance, H_2_O_2_ at sufficiently high concentrations can pass through cell membranes and cause oxidative stress, mutagenesis and the bleaching of chlorophy II^15–17^. Therefore, it is not surprising that research on H_2_O_2_ has been carried out by numerous investigators, especially the development of its detection techniques which has profound implication on estimating net production of H_2_O_2_ in natural waters and our understanding of ecosystem biogeochemistry.

Up to now, more than 30 analytical methods based on absorbance, voltammetry, fluorescence or chemiluminescence have been developed to determine H_2_O_2_ distribution in various aquatic environment such as surface waters^18^. Each method attempts to accelerate the analysis process, reduce the detection limits and avoid potential interferences such as fluorescence quenching by DOM^19^. Among these methods, fluorescence (FL) method is by far the most popular one, where the probe compound is oxidized to yield products that either exhibit FL or whose FL is diminished in the presence of peroxidase and H_2_O_2_^18^. The most commonly used and highly cited FL method for determination of H_2_O_2_ in natural waters in early studies was scopoletin-horseradish peroxidase (HRP) method^18^. Although no longer the most commonly used method for the quantitative determination of H_2_O_2_, it is the seminal method from which many current methods evolved and so a presentation of some methodological detail is appropriate for any review^18^. Currently, the method have been demonstrated to suffer from the potential interference from various factors including naturally occurring substances such as DOM and organic peroxide, pH and buffer^2,18,20,21^. For example, Cooper et al. (1988) determined H_2_O_2_ concentrations in natural water matrices including freshwater, agricultural water, sewage, seawater and estuarine exposed to sunlight using scopoletin method. The samples were prepared in phosphate buffer (PBS) adjusted to pH 7 and diluted if their total organic carbon (TOC) of was higher than 2 mgC L^−1^ prior to analysis. Miller and Kester (1988) discussed interferences of DOM and organic peroxide to H_2_O_2_ determination using another HRP-mediated (*p*-Hydroxyphenyl) acetic acid (POHPAA) method, and recommended using standard additions to determine H_2_O_2_ in natural water samples to overcome DOM interferences. Afterward, standard addition method has been widely applied in H_2_O_2_ determination in natural waters^22^. In fact, both the aqueous and soil environments contain large amounts of natural phenols (e.g., ferulic acid, syringaldehyde, pyrogallol, hydroxybenzoic acid, or catechol) that originate from lignin decomposition and are major substrates for oxidative coupling reaction leading to the formation of humus^23^. In addition, some xenobiotic compounds, e.g., chlorinated phenols and anilines, were introduced into the environment by accidental spills, illegal release of industrial and municipal wastewater, and excessive use of pesticides, and were regarded as analogues of DOM constituents^24^. As DOM constituents with phenolic, amino, and/or aromatic alcohols moiety as the core components in their chemical structures were also the excellent substrates of peroxidases, it is speculated that DOM constituents may interfere the measurement of H_2_O_2_ by peroxidase-mediated depletion of scopoletin fluorescence method. However, with the exception of DOM and organic peroxide, those heretofore neglected or overlooked other interferences in the determination of H_2_O_2_ in natural waters should be of concern. In view of this idea, knowledge related to the effect of DOM constituents on the determination of H_2_O_2_ in waters is of great environmental significance.

The aims of the present study were (1) to investigate and compare the influence of 6 representing forms of DOM and its constituents on the determination of environmental level H_2_O_2_ using the HRP-mediated depletion of scopoletin fluorescence method. In light of the diversity of specific DOM constituents (Fig. S1), 24 compounds with phenolic, carbonyl, carboxyl, and amino groups attached to benzene skeleton were selected; (2) to establish a quantitative structure–activity relationship (QSAR) model based on the data of interfering effect of DOM constituents on H_2_O_2_ concentration determination with good stability and prediction ability, thereby trying to predict the effect of other DOM constituent analogues on the H_2_O_2_ measurement in water.

## Results and discussion

### Interference of DOM on H_2_O_2_ determination

**Figure 1:**
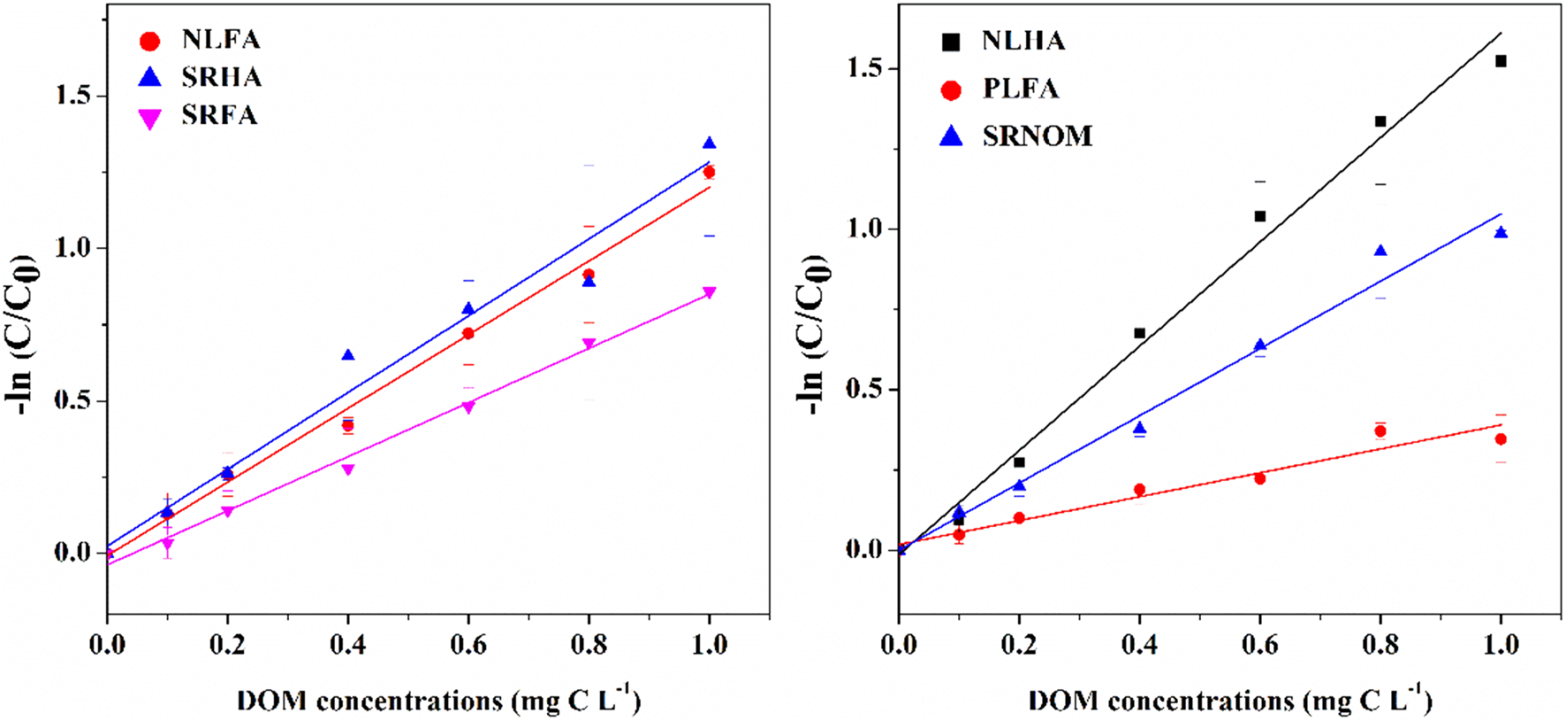
The effect of DOM originated from different sources on H_2_O_2_ determination.

**Figure 2:**
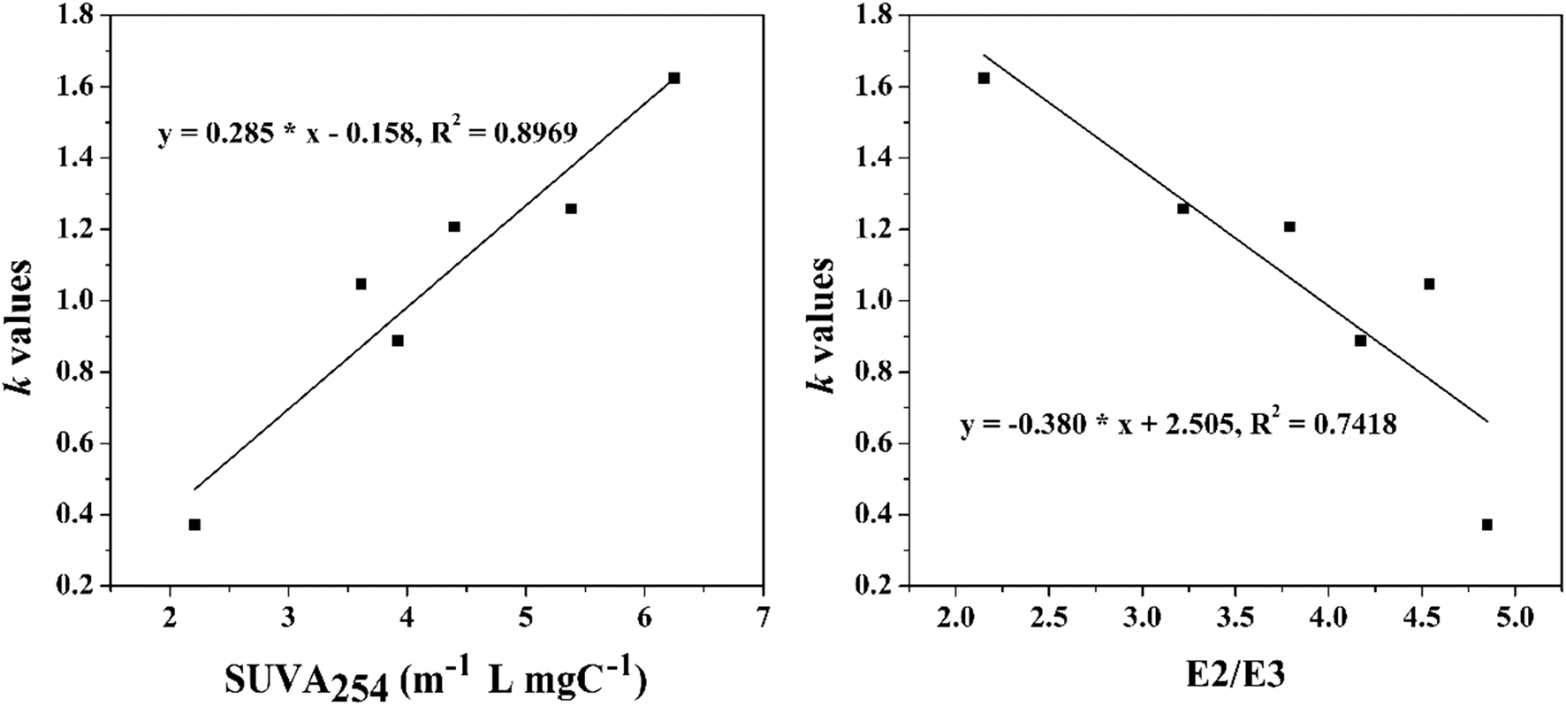
The relationship between SUVA_254_ and E2/E3 and the *k* of DOM.

The effects of 6 kinds of representative DOM on H_2_O_2_ determination were presented in Fig. 1. For each investigated DOM, it was apparent that DOM significantly interfere the accurate measurement of H_2_O_2_ content, and the measured concentrations were lower than the nominal concentration (200 nM). To be clear, here the decreasing ratio (C/C_0_) of measured H_2_O_2_ concentration caused by the presence of DOM was defined as the ratio of measured concentration of H_2_O_2_ (experimental values, C) and initial concentration of H_2_O_2_ (theoretical values, C_0_). Figure 2 displayed the relationship between each DOM concentration and the corresponding decreasing ratio to H_2_O_2_. As can be seen, the determinable H_2_O_2_ concentration became decreased with the increasing contents of DOM. There is a fine linear relationship between DOM concentrations and the natural logarithm of decreasing ratios (R^2^ > 0.9), thus the obtained slope *k* ((mgC L^−1^)^−1^) values were called as interfering ratios, expressing the interfering degree of DOM per unit concentration to the measurement of H_2_O_2_, and the greater the *k* values, the larger the interfering degree of DOM to H_2_O_2_ measurement. The interfering ratios of DOM from different sources and types were showed in Table 1. The interfering ratio of HA from Nordic lake (NLHA) was the highest, and significantly higher than SRFA, PLFA and SRNOM (*p* < 0.05). The interfering ratio of FA from Pony lake (PLFA) is significantly lower than NLFA, SRHA, NLHA and SRNOM (*p*  < 0.05) (Table S1). The parameter EC_10_ (mgC L^−1^) in Table 1 expressed DOM concentrations added when the decreasing ratio of measured H_2_O_2_ concentration reach to 10%, which could be considered as not significant. As showed in Table 1, the EC_10_ values of most FAs were greater than 0.1 mgC L^−1^, and generally greater than that of HAs. On the whole, accurate determination of H_2_O_2_ using peroxidase-mediated depletion of scopoletin fluorescence method will be disturbed substantially when DOM levels exceed 0.1 mgC L^−1^ in natural waters. In fact, the level of DOM in actual water bodies usually ranged from a few to tens of mgC L^−1^^7,25^. As a result, the water samples should be diluted by tens to hundreds of times to accurately determine H_2_O_2_ levels in natural waters.

**Table 1:** The fitted equation of interfering ratio of DOM on H_2_O_2_ determination and DOM concentration. *k* expressed the interfering degree of DOM per unit concentration to the measurement of H_2_O_2_ and EC_10_ expressed DOM concentrations added when the decreasing ratio of measured H_2_O_2_ concentration reach to 10%.

DOM	Fitted equations	R^2^	*k* ((mgC L^−1^)^−1^)	EC_10_ (mgC L^−1^)
NLFA	y = 1.198 * x	0.9968	1.198	0.088
SRHA	y = 1.293 * x	0.9871	1.293	0.084
SRFA	y = 0.836 * x	0.9945	0.836	0.126
NLHA	y = 1.606 * x	0.9953	1.606	0.066
PLFA	y = 0.398 * x	0.9764	0.398	0.265
SRNOM	y = 1.048 * x	0.9932	1.048	0.101

The SUVA_254_ determined the absorbance at 254 nm divided by DOM concentration and E2/E3 determined the absorbance at 254 nm divided by the absorbance at 365 nm are important indicators that reflect the aromaticity and molecular weight of DOM, respectively^26^. Here the SUVA_254_ and E2/E3 of investigated six DOM at environmentally relevant concentration of 5 mgC L^−1^ were calculated in Table S2, and the relationship between them and the *k* of DOM was plotted in Fig. 2. As can be seen, the *k* of DOM displayed a negative correlation with E2/E3 values, and a good positive correlation with their SUVA_254_ values, which could be explained that DOM with the larger molecular weight and aromaticity may wrap the substrate, and thus prevent it from contacting HRP^26^. Based on our finding, the accurate determination of H_2_O_2_ in natural waters by a peroxidase-mediated depletion of scopoletin fluorescence method could be corrected according to the measurement of SUVA_254_ and E2/E3 of DOM. Our results would also provide information on the reliability evaluation of the interference of DOM on the determination of H_2_O_2_.

### Influence of DOM constituents on H_2_O_2_ determination

**Figure 3:**
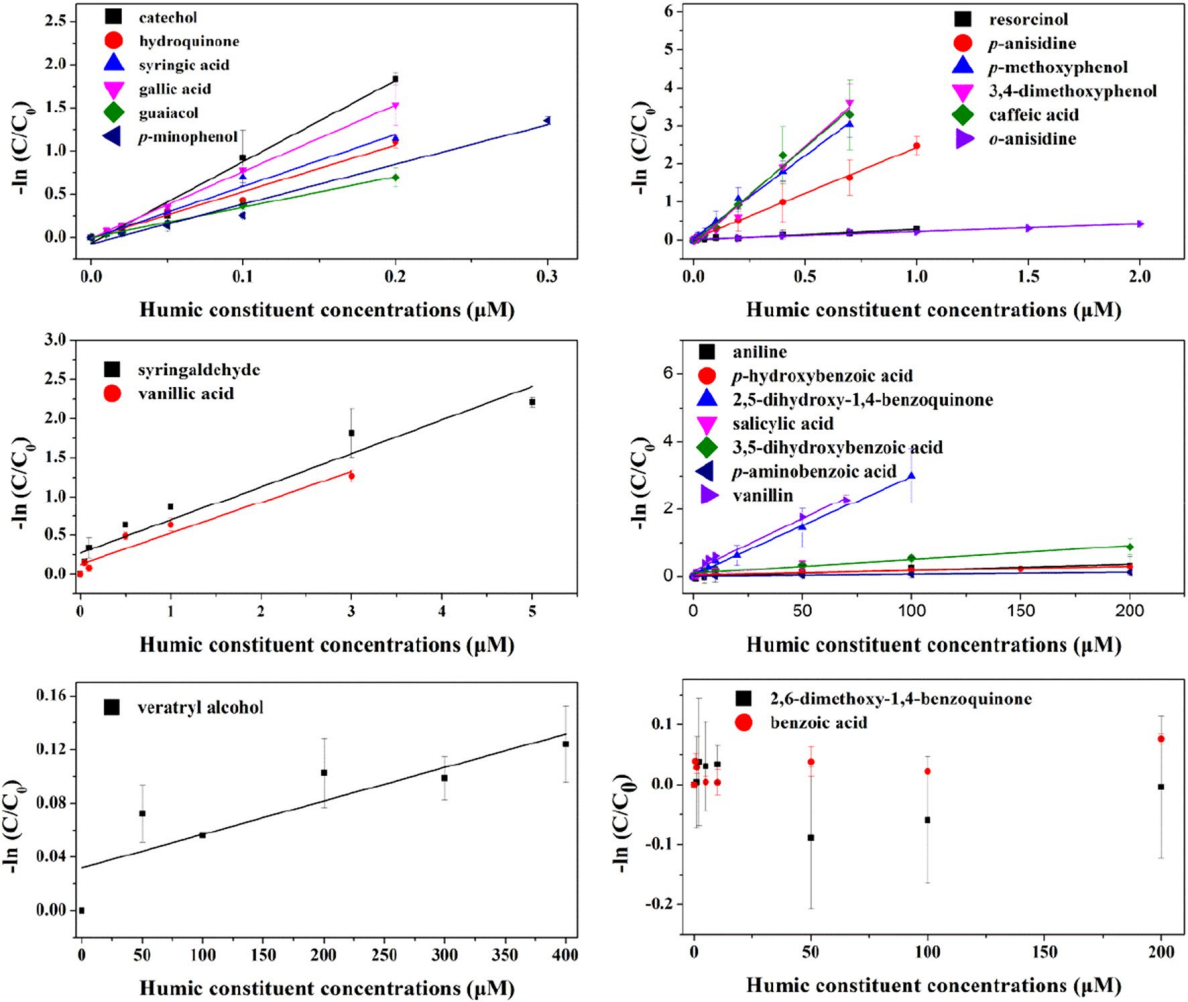
The effects of natural DOM constituents on H_2_O_2_ determination.

Similarly, impacts of 24 DOM constituents on H_2_O_2_ at the initial concentration of 200 nM were explored, and the results were showed in Fig. 3. In general, most compounds interfered the measurement of H_2_O_2_ in different degree, and there is good linear relationship between compound concentrations and the natural logarithm of decreasing ratios (R^2^ > 0.9) with the exception of 2,6-dimethoxy-1,4-benzoquinone and benzoic acid. For 2,6-dimethoxy-1,4-benzoquinone, the fluorescence signal became weaker with its concentration increasing, which was probably explained by the quenching effect of the compound on the fluorescence intensity of scopoletin. As for benzoic acid, there was no obvious interference for H_2_O_2_ measurement regardless of its concentration.

Table 2 listed the decreasing ratios of the DOM constituents on H_2_O_2_ determination. Except for 2,6-dimethoxy-1,4-benzoquinone and benzoic acid, the order of magnitude of interfering ratios of all other compounds take on a large span from 10^–3^ to 10^–7^ μM^-1^. In a word, catechol gave rise to the maximum interfering on H_2_O_2_ measurement and was significantly higher than that of all compounds with the interference for H_2_O_2_ measurement except for gallic acid (*p* < 0.05, Table S3). There was significant effect on measurement of H_2_O_2_ at 200 nM level when the concentrations of those 5 compounds reached to 20 nM in waters, and measurable H_2_O_2_ concentration can be completely suppressed when their concentrations increased to 200 nM. H_2_O_2_ determination will be disturbed by guaiacol, *p*-anisidine, 3,4-dimethoxyphenol, *p*-aminophenol and caffeic acid at 30 nM, and may be submerged absolutely on the condition of 700 nM of the same compounds. As for resorcinol and o-anisidine, when their concentration came up to 200 and 400 nM respectively, H_2_O_2_ measurement would be affected, and be completely suppressed at 5 and 7 μM of the 2 compounds respectively. The ones induced the weakest interfering effect on H_2_O_2_ were *p*-hydroxybenzoic acid, 2,5-dihydroxy-1,4-benzoquinone, aniline, *p*-aminobenzoic acid, salicylic acid and veratryl alcohol. The significant impact on H_2_O_2_ measurement required the levels of these compound reaching up to several μM, and even hundreds of μM.

**Table 2:** The fitted equation of interfering ratios of DOM constituents on H_2_O_2_ and DOM constituent concentrations. *k* expressed the interfering degree of DOM constituents per unit concentration to the measurement of H_2_O_2_ and EC_10_ expressed DOM constituent concentrations added when the decreasing ratio of measured H_2_O_2_ concentration reach to 10%.

DOM constituents	Fitted equations	R^2^	*k* (μM^−1^)	EC_10_ (nM)
Catechol	y = 8.955 * x	0.9877	8.955	12
Resorcinol	y = 0.282 * x	0.9760	0.282	374
Hydroquinone	y = 5.320 * x	0.9886	5.320	20
Guaiacol	y = 3.505 * x	0.9983	3.505	30
*p*-methoxyphenol	y = 4.442 * x	0.9958	4.442	24
3,4-Dimethoxyphenol	y = 4.913 * x	0.9836	4.913	21
*p*-Aminophenol	y = 4.260 * x	0.9722	4.260	25
Benzoic acid	–	–	–	–
Syringic acid	y = 5.932 * x	0.9891	5.932	18
Gallic acid	y = 7.645 * x	0.9993	7.645	14
*p*-Hydroxybenzoic acid	y = 0.00167*x	0.9244	0.00167	63,090
Salicylic acid	y = 0.00634 * x	0.8511	0.00634	16,618
3,5-Dihydroxybenzoic acid	y = 0.00481 * x	0.9176	0.00481	21,904
Caffeic acid	y = 4.862 * x	0.9904	4.862	22
*p*-Aminobenzoic acid	y = 0.000698 * x	0.9496	0.000698	150,948
Vanillic acid	y = 0.458 * x	0.9328	0.458	230
Vanillin	y = 0.0344 * x	0.9711	0.0344	3063
Syringaldehyde	y = 0.503 * x	0.9302	0.503	209
Aniline	y = 0.00193* x	0.8894	0.00193	54,591
*o*-Anisidine	y = 0.215* x	0.9852	0.215	529
*p*-Anisidine	y = 2.443 * x	0.9987	2.443	43
2,5-Dihydroxy-1,4-benzoquinone	y = 0.0301 * x	0.9953	0.0301	3500
2,6-Dimethoxy-1,4-benzoquinone	–	–	–	–
Veratryl alcohol	y = 0.000360 * x	0.8705	0.000360	292,668

Different inhibition ratios of DOM constituents may be connected with their functional group type, number and position of these compounds. For instance, in terms of isomers, the interfering effect of catechol to H_2_O_2_ measurement was close to that of hydroquinone, while was higher than that of resorcinol. Moreover, guaiacol *versus p*-methoxyphenol, and salicylic acid *versus p*-hydroxybenzonic acid have presented a parallel effect. For non-isomers with the same substituent position, type and number, a good agreement on the interfering impact existed between catechol and hydroquinone with -OH *versus* guaiacol and *p*-methoxyphenol with -OCH_3_, respectively, which was similar to hydroquinone *versus p*-aminophenol and *p*-methoxyphenol *versus p*-anisidine. A comparison for the interfering effect of hydroquinone *versus p*-hydroxybenzoic acid, catechol *versus* salicylic acid, and *p*-aminobenzoic acid, *p*-anisidine and *p*-aminophenol might indicate that -COOH caused a slight action on H_2_O_2_ determination.

These results were significant to determine precisely the levels of H_2_O_2_ in natural waters. Montero et al. detected phenol and several chlorophenols (e.g., 2-chlorophenol and 2,4-dimethylphenol) concentration ranged from ng L^−1^ to μg L^−1^ in lakes and ground waters, and the total amount of analyzed phenols ranged from 43 to 138 μg L^−1^^27^. Davi et al. have found phenolic compounds (e.g., nonylphenol and alkylphenol) with the concentration of up to tens of μg L^−1^ in river Po water which is utilized to produce drinking water^28^. In coast of Thermaikos Gulf, Northern Greece, various nitro- and chlorophenols were monitored from 2003 to 2004, and the maximum concentration of chlorophenols were observed for pentachlorophenol (8.04 μg L^−1^) followed by 2,4-dichlorophenol (6.11 μg L^−1^)^29^. The levels of the mentioned phenolic compounds are in the range of nM to μM when their mass concentration was converted to molar concentration. In context, the current levels of these compounds in natural waters did interfere with the determination of H_2_O_2_ on the basis of given 200 nM H_2_O_2_ in our experiment, resulting in dramatically underestimating the actual concentration of H_2_O_2_ in the aquatic environment.

### Relationship between the log *k* of DOM constituents and their molecular descriptors

**Figure 4:**
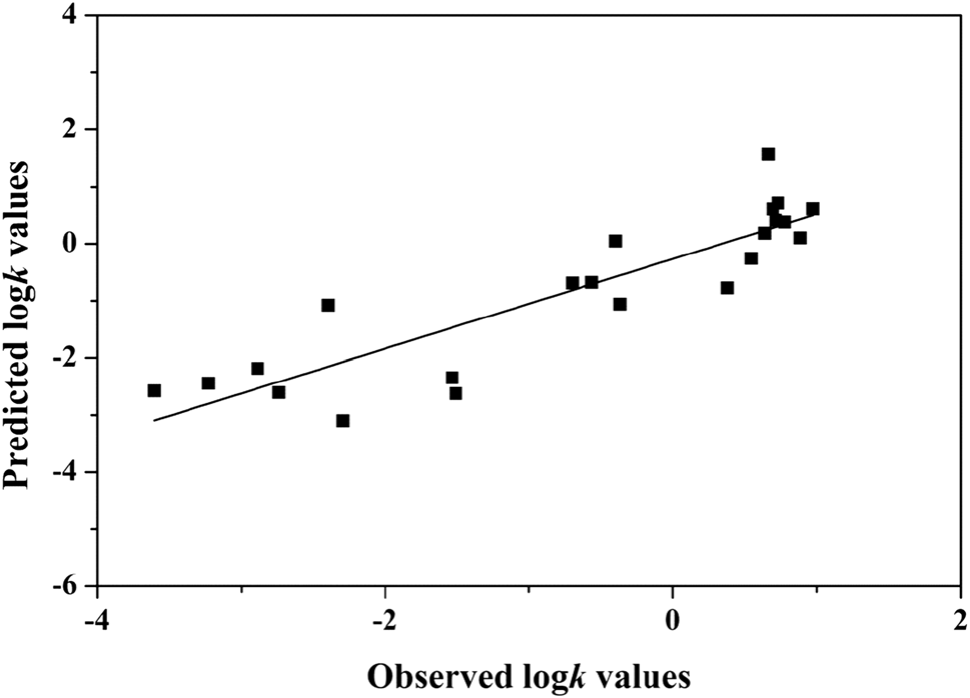
Plot of predicted versus observed values of log *k*.

We selected 22 compounds having significant interfering effect on H_2_O_2_ measurement as target, and tried to build a model between the obtained *k* and the calculated 14 physicochemical and quantum-chemical descriptors of the corresponding compound by stepwise multiple linear regression method embodied in SPSS 17.0. The resulting four-parameter optimal equation was obtained as following (Eq. 1):

1 $$\begin{aligned} {\text{Log}}k & = 15.115 + 106.{\text{915E}}_{{{\text{HOMO}}}} + 19.{\text{648qH}}^{ + } - 0.0{\text{22H }} - 0.{\text{118V}} \\ {\text{n}} & = 22\quad {\text{R }} = 0.886\quad {\text{SE }} = 0.684\quad {\text{q }} = 0.799\quad {\text{F }} = 15.54 \\ \end{aligned}$$

where n, R, SE, q and F represent the number of compounds, the correlation coefficient, the standard error, the leave-one-out cross validation coefficient, and the Fisher test value of the equation, respectively. The robustness and internal predictive power of the model (Eq. 1) were assessed according to q^2^. Here, q^2^ (q^2^ = 0.638) is greater than 0.5, indicating that the model has good robustness and predictive power. The F value of Eq. (1) (F = 15.54) is greater than the critical value at the confidence levels of 95% (F_0.05_ = 2.965), indicating that the model has statistical significance. Equation (1) has high R and low SE values. Thus, all of the correlative relationships are significant, suggesting that using molecular descriptors to fit the log *k* of natural DOM constituents is successful. The four parameters E_homo_, qH^+^, H and V were taken into Eq. (1). It can be seen from Eq. (1) that log *k* value was positively correlated with E_homo_ and qH^+^, and negatively with H and V. The reasons may be that (1) the compounds with higher E_homo_ have the stronger electron donor capacity, and more easily replace scopoletin to react with phenolic radical, (2) the compounds with the larger qH^+^ have the stronger electron-withdrawing capacity, resulting in readily forming hydrogen bond with water and going into the water phase to react, (3) H is related to the stability of compound. A compound with larger H is more stable, and is hard to take part in reaction, (4) V is associated with the hydrophobicity of a compound. The compound with the larger V is more prone to distribute to the organic phase. Figure 4 displayed the plot of the predicted *versus* observed log *k* values. As can be seen, the established model has good linear feature and high predictive power.

## Conclusions

This study demonstrated that DOM and its constituents such as catechol, gallic acid, syringic acid in waters could significantly suppress the measured amounts of H_2_O_2_ by using a peroxidase-mediated depletion of scopoletin fluorescence method, and the interfering extents were parallel for DOM and varied vastly for DOM constituents. The results will provide new insight into the accurate determination of H_2_O_2_ in natural waters, whereby implicating its environmental behavior. Based on the 14 physicochemical and quantum-chemical descriptors of 22 DOM constituents, a QSAR model with remarkable stability and strong predictability for evaluating the interfering ratio of DOM constituents to H_2_O_2_ measurement was established by multiple linear regression method, which could be used to predict the effect of DOM constituent analogues on H_2_O_2_ determination. Nevertheless, further works are needed to better understand the specific effect of DOM and its constituents on H_2_O_2_ measurement in situ in the future in light of the complexity of natural waters.

## Methods

### Chemicals

H_2_O_2_ (w/w 30%), phenol (99%), scopoletin (99%) and HRP (type II, EC 1.11. 1.7) were obtained from Sigma-Aldrich (St. Louis, MO). 24 kinds of natural DOM constituents including catechol (99%), resorcinol (99%), hydroquinone (99%), guaiacol (99%), *p*-methoxyphenol (99%), 3,4-dimethoxyphenol (99%), *p*-aminophenol (99%), vanillin (99%), syringaldehyde (99%), aniline (99%), o-anisidine (99%), *p*-anisidine (99%), benzoic acid (> 99%), syringic acid (99%), gallic acid (99%), *p*-hydroxybenzoic acid (99%), salicylic acid (99%), 3,5-dihydroxybenzoic acid (> 97%), caffeic acid (99%), *p*-aminobenzoic acid (99%), vanillic acid (99%), 2,5-dihydroxy-1,4-benzoquinone (99%), 2,6-dimethoxy-1,4-benzoquinone (99%) and veratryl alcohol (> 98%) were purchased from J & K Scientific Ltd. (Shanghai, China), and the corresponding molecular weight and structural formula were shown in Table S4. All the reagents were of high performance liquid chromatography (HPLC) grade or higher and used as received unless otherwise stated. Six forms of DOM containing Suwannee Rive Humic Acid (SRHA), Suwannee River Fulvic Acid (SRFA), Suwannee River Natural Organic Matter (SRNOM), Nordic Lake Fulvic Acid (NLFA), Nordic Lake Humic Acid (NLHA) and Pony Lake Fulvic Acid (PLFA) were purchased from International Humic Substance Society (IHSS), and the corresponding C, H, O content were displayed in Table S5.

A 50 mgC L^−1^ stock solution of each DOM measured by a Shimadzu TOC-L analyzer and the quantification was based on a standard calibration of potassium dihydrogen phthalate solution in the range of 1–20 mg L^−1^. 1 mM stock solution of each DOM constituent were prepared in ultrapure water obtained from Milli-Q purification system. The working standard solution of 20 μM H_2_O_2_ was prepared daily by dilution of 30% H_2_O_2_ stock solution calibrated via a titrimetric method using potassium permanganate titration solution just before use^30^.

### Experimental procedures

All experiments were carried out in 10 mL colorimetric tubes. Each reaction solution was prepared in 0.01 M phosphate buffer solution (PBS) (pH 7.0), consisting of H_2_O_2_ with an initial concentration of 200 nM and a DOM or its constituent with a series of prescribed levels. It was noticed that the regulated concentration of H_2_O_2_ was a typical level in natural water^2,31^. A 8 mL sample was withdrawn from each tube in quadruplicate to a 5 mL Teflon tubes. Subsequently, an 80 μL of 5 × 10^–6^ M scopoletin was added to one of the tubes for 5 min, and then a 40 μL volume of the working solution containing 12.5 U mL^−1^ HRP and 1 mM phenol in 0.01 mM PBS (pH 7.0) was also added. After shaken for 5 min at room temperature, the mixture was analyzed in a 1 cm cuvette using a fluorometric method to determine the apparent concentration of H_2_O_2_. The remaining three samples were used to determine blanks. In detail, Blanks were analyzed to quantify the fluorescence response of catalase, the fluorometric reagent, and the sample solution. The catalase blank was determined by decomposing the aqueous H_2_O_2_ and organic peroxides in one sample: 50 μL of 2.0 U mL^−1^ catalase was added to each tube followed by a 5 min reaction time (CAT blank). Similarly, after addition of catalase for 5 min, 40 μL fluorometric reagent was added one of the remaining two tubes, and the reaction was allowed to proceed for an additional 5 min (FL blank). The last one sample acted as the natural blank of reaction solution (NAT blank). After fluorometric analysis of these blanks, the total blank was calculated from the following relationship:

$${\text{Total}}\;{\text{blank }} = {\text{ NAT}} + \left( {{\text{FL}}{-}{\text{CAT}}} \right)$$

Note, catalase was added to one of the samples prior to the fluorescence reagent to test whether the observed signal was arising from H_2_O_2_.

### H_2_O_2_ analyses

The concentration of H_2_O_2_ was measured by a HRP-mediated depletion of scopoletin fluorescence using a FluoroMax-4 fluorescence spectrometer (Horiba, France) with excitation centered at 354 nm and emission centered at 496 nm. The sampling intervals on excitation and emission modes were both set at 1 nm. The fluorometer was periodically calibrated with 100 nM quinine sulfate in 0.05 M sulfuric acid. Prior to calibration, the fluorometer was zeroed with ultrapure water. Decrease in the magnitude of the fluorescence signal was converted to concentrations of reacted H_2_O_2_ employing a calibration curve. The 8 mL of H_2_O_2_ standard solution was prepared in 0.01 mM PBS (pH 7.0) and in quadruplicate to 5 mL Teflon tubes, and the following analysis procedure is the same as above. Three blanks, i.e., CAT blank, FL blank and NAT blank, were parallelly performed. Linear ranges of standard operating curves for H_2_O_2_ concentration were from 10 to 500 nM. The detection limit, defined as 3 times the standard deviation of the blanks, was 10 nM.

### Characterization of DOM and its constituents

The 5 mgC L^−1^ of each DOM and its constituent was prepared by dilution of the corresponding stock solution for characterization of UV–Vis spectra. The process was carried out with 1 cm quartz cuvette in Varian Cary 100 scan UV–Vis spectrophotometer, and the absorbance of each DOM at 254 nm and 365 nm was acquired, respectively.

### Calculation of the molecular parameters

The structural and thermodynamic parameters of DOM constituents in water solution were computed by density functional theory (DFT) method and Onsager model in self-consistent field (SCRF) at the DFT/B3LYP/6-31G** level. The resulting structural and thermodynamic parameters including energy of the lowest unoccupied molecular orbital (E_lumo_), energy of the highest occupied molecular orbital (E_homo_), dipole moment (*μ*), the most positive net charge of hydrogen atoms (qH^+^), the most negative atomic net charge of molecule (*q*^-^), total energy (TE), molecular average polarizability (*α*), zero-point vibration energy (ZPE), Gibbs free energy (G^θ^), enthalpy (H^θ^), entropy (S^θ^), corrected heat energy (E_th_), molecular volume (V), and molar heat capacity at constant volume (C_v_^θ^) were used to build QSAR models by the SPSS version 17.0 (Table S6). With the propose of obtaining optimum number of variables for the correlation model, stepwise multiple linear regression procedure was employed to establish the dependent equation by adopting structural and thermodynamic parameters calculated at the B3LYP/6-311G** level. The model stability was validated by variance analysis and standard regression coefficient.

### Statistical analysis

All data were presented as means ± SD (standard deviation) from triplicated experiments. Intergroup differences were assessed using one-way analysis of variance (ANOVA) based on Tukey's multiple comparisons test by SPSS statistical package (ver. 16.0, SPSS Company, Chicago, USA). Differences between groups were considered as statistically significant if *p* < 0.05.

## Supplementary Information

Supplementary Information.
