# Supplementary material for: Interference of dissolved organic matter and its constituents on the accurate determination of hydrogen peroxide in water
Source: Sci Rep. 2021 Nov 19;11:22613. doi: 10.1038/s41598-021-01016-9 (PMC8604898; doi:10.1038/s41598-021-01016-9)
Supplement: Supplementary file 1 — Supplementary Information. [file 41598_2021_1016_MOESM1_ESM.docx]

**Interference of dissolved organic matters and its constituents on the accurate determination of hydrogen peroxide in water**

Jianbiao Peng^2^, Ya Zhang^1^, Jianhua Li^3^, Xinan Wu^4^, Mengjie Wang^1^, Zhimin Gong^4^ & Shixiang Gao^4^

Number of Pages: 8

Number of Figures: 1

Number of Tables: 6

Nanjing Institute of Environmental Sciences, Ministry of Environmental Protection of the People’s Republic of China, Nanjing 210042, P.R. China. 2School of Environment, Henan Normal University, Key Laboratory for Yellow River and Huai River Water Environmental and Pollution Control, Ministry of Education, Henan Key Laboratory for Environmental Pollution Control, Xinxiang 453007, P.R. China. 3Jiangsu Key Laboratory of Chemical Pollution Control and Resources Reuse, School of Environmental and Biological Engineering, Nanjing University of Science and Technology, Nanjing 210094, P.R. China. 4State Key Laboratory of Pollution Control and Resource Reuse, School of the Environment, Nanjing University, Nanjing 210023, P.R. China. Correspondence and requests for materials should be addressed to Y.Z. ([zhangya@nies.org](mailto:zhangya@nies.org))

Fig. S1 A proposed structure for humic acid ([Clark et al., 2014](#_ENREF_1)).

Table S1 Significant analysis among DOM

|  | NLFA | SRHA | SRFA | NLHA | PLFA | SRNOM |
| --- | --- | --- | --- | --- | --- | --- |
| NLFA | — | — | — | — | — | — |
| SRHA | No^a^ | — | — | — | — | — |
| SRFA | No | No | — | — | — | — |
| NLHA | No | No | Yes** | — | — | — |
| PLFA | Yes**^b^ | Yes** | No | Yes***^c^ | — | — |
| SRNOM | No | No | No | Yes*^d^ | Yes* | — |

^a^ No: No significant difference, p > 0.05, ^b^**: Significant difference, p < 0.01, ^c^ ***: Significant difference, p < 0.001, ^d^*: Significant difference, p < 0.05.

Table S2 Characterization of DOM

| DOM | UVA_254_ (cm^-1^) | SUVA_254_ ^a^(m^-1^ L mgC^-1^) | UVA_365_ (cm^-1^) | E2/E3^b^ |
| --- | --- | --- | --- | --- |
| PLFA | 0.1106 | 2.21 | 0.0228 | 4.85 |
| SRNOM | 0.1805 | 3.61 | 0.0398 | 4.54 |
| SRFA | 0.1961 | 3.92 | 0.0470 | 4.17 |
| NLFA | 0.2201 | 4.40 | 0.0580 | 3.79 |
| SRHA | 0.2692 | 5.38 | 0.0837 | 3.22 |
| NLHA | 0.3125 | 6.25 | 0.1453 | 2.15 |

^a^ SUVA_254_ = UVA_254_/DOM concentration, ^b^ E2/E3 = UV_254_/UV_365_.

Table S3 Significant analysis among 22 DOM constituents

|  | **catechol** | **resorcinol** | **hydroquinone** | **guaiacol** | ***p*-methoxyphenol** | **3,4-dimethoxyphenol** | ***p*-aminophenol** | **syringic acid** | **gallic acid** | ***p*-hydroxybenzoic acid** | **salicylic acid** | **3,5-dihydroxybenzoic acid** | **caffeic acid** | ***p*-aminobenzoic acid** | **vanillic acid** | **vanillin** | **syringaldehyde** | **aniline** | ***o*-anisidine** | ***p*-anisidine** | **2,5-dihydroxy-1,4-benzoquinone** | **veratryl alcohol** |
| --- | --- | --- | --- | --- | --- | --- | --- | --- | --- | --- | --- | --- | --- | --- | --- | --- | --- | --- | --- | --- | --- | --- |
| **catechol** | — | — | — | — | — | — | — | — | — | — | — | — | — | — | — | — | — | — | — | — | — | — |
| **resorcinol** | Yes****^a^ | — | — | — | — | — | — | — | — | — | — | — | — | — | — | — | — | — | — | — | — | — |
| **hydroquinone** | Yes**** | Yes**** | — | — | — | — | — | — | — | — | — | — | — | — | — | — | — | — | — | — | — | — |
| **guaiacol** | Yes**** | Yes*** | No | — | — | — | — | — | — | — | — | — | — | — | — | — | — | — | — | — | — | — |
| ***p*-methoxyphenol** | Yes**** | Yes**** | No | No | — | — | — | — | — | — | — | — | — | — | — | — | — | — | — | — | — | — |
| **3,4-dimethoxyphenol** | Yes**** | Yes**** | No | No | No | — | — | — | — | — | — | — | — | — | — | — | — | — | — | — | — | — |
| ***p*-aminophenol** | Yes**** | Yes**** | No | No | No | No | — | — | — | — | — | — | — | — | — | — | — | — | — | — | — | — |
| **syringic acid** | Yes***^b^ | Yes**** | No | Yes** ^e^ | No | No | No | — | — | — | — | — | — | — | — | — | — | — | — | — | — | — |
| **gallic acid** | No ^c^ | Yes**** | Yes*^d^ | Yes**** | Yes*** | Yes** | Yes**** | No | — | — | — | — | — | — | — | — | — | — | — | — | — | — |
| ***p*-hydroxybenzoic acid** | Yes**** | No | Yes**** | Yes**** | Yes**** | Yes**** | Yes**** | Yes**** | Yes**** | — | — | — | — | — | — | — | — | — | — | — | — | — |
| **salicylic acid** | Yes**** | No | Yes**** | Yes**** | Yes**** | Yes**** | Yes**** | Yes**** | Yes**** | No | — | — | — | — | — | — | — | — | — | — | — | — |
| **3,5-dihydroxybenzoic acid** | Yes**** | No | Yes**** | Yes**** | Yes**** | Yes**** | Yes**** | Yes**** | Yes**** | No | No | — | — | — | — | — | — | — | — | — | — | — |
| **caffeic acid** | Yes**** | Yes**** | No | No | No | No | No | No | Yes** | Yes**** | Yes**** | Yes**** | — | — | — | — | — | — | — | — | — | — |
| ***p*-aminobenzoic acid** | Yes**** | No | Yes**** | Yes**** | Yes**** | Yes**** | Yes**** | Yes**** | Yes**** | No | No | No | Yes**** | — | — | — | — | — | — | — | — | — |
| **vanillic acid** | Yes**** | No | Yes**** | Yes*** | Yes**** | Yes**** | Yes**** | Yes**** | Yes**** | No | No | No | Yes**** | No | — | — | — | — | — | — | — | — |
| **vanillin** | Yes**** | No | Yes**** | Yes**** | Yes**** | Yes**** | Yes**** | Yes**** | Yes**** | No | No | No | Yes**** | No | No | — | — | — | — | — | — | — |
| **syringaldehyde** | Yes**** | No | Yes**** | Yes*** | Yes**** | Yes**** | Yes**** | Yes**** | Yes**** | No | No | No | Yes**** | No | No | No | — | — | — | — | — | — |
| **aniline** | Yes**** | No | Yes**** | Yes**** | Yes**** | Yes**** | Yes**** | Yes**** | Yes**** | No | No | No | Yes**** | No | No | No | No | — | — | — | — | — |
| ***o*-anisidine** | Yes**** | No | Yes**** | Yes*** | Yes**** | Yes**** | Yes**** | Yes**** | Yes**** | No | No | No | Yes**** | No | No | No | No | No | — | — | — | — |
| ***p*-anisidine** | Yes**** | Yes* | Yes**** | No | Yes* | Yes** | No | Yes**** | Yes**** | Yes** | Yes** | Yes** | Yes**** | Yes** | Yes* | Yes** | No | Yes** | Yes* | — | — | — |
| **2,5-dihydroxy-1,4-benzoquinone** | Yes**** | No | Yes**** | Yes**** | Yes**** | Yes**** | Yes**** | Yes**** | Yes**** | No | No | No | Yes**** | No | No | No | No | No | No | Yes** | — | — |
| **veratryl alcohol** | Yes**** | No | Yes**** | Yes**** | Yes**** | Yes**** | Yes**** | Yes**** | Yes**** | No | No | No | Yes**** | No | No | No | No | No | No | Yes** | No | — |

^a^ ****: Significant difference, p < 0.0001, ^b^ ***: Significant difference, p < 0.001, ^c^ No: No significant difference, p > 0.05, ^d^ *: Significant difference, p < 0.05, ^e^ **: Significant difference, p < 0.01.

Table S4 DOM constituents

| **phenols** | | | **benzoic acids** | | |
| --- | --- | --- | --- | --- | --- |
| catechol | 110.11 | 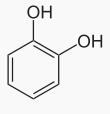 | benzoic acid | 122.12 | 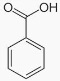 |
| resorcinol | 110.11 | 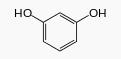 | syringic acid | 198.18 | 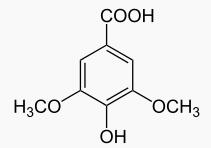 |
| hydroquinone | 110.11 | 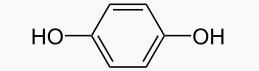 | gallic acid | 170.19 | 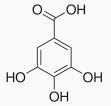 |
| guaiacol | 124.14 | 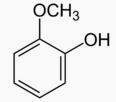 | *p*-hydroxybenzoic acid | 138.13 |  |
| *p*-methoxyphenol | 124.14 | 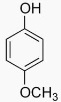 | salicylic acid | 138.12 | 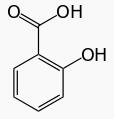 |
| 3,4-dimethoxyphenol | 154.16 |  | 3,5-dihydroxybenzoic acid | 154.12 | 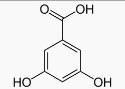 |
| *p*-aminophenol | 109.13 |  | caffeic acid | 180.15 |  |
| **aldehydes** | | | *p*-aminobenzoic acid | 137.14 | 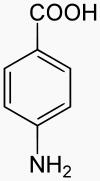 |
| vanillin | 152.14 | **** | vanillic acid | 168.15 |  |
| syringaldehyde | 182.17 | **** | **benzoquinones** | | |
| **anilines** | | | 2,5-dihydroxy-1,4-benzoquinone | 140.09 | 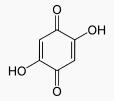 |
| aniline | 93.14 | 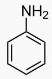 | 2,6-dimethoxy-1,4-benzoquinone | 168.15 | 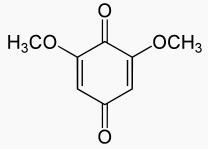 |
| *o*-anisidine | 123.15 | 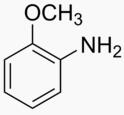 | **aromatic alcohols** | | |
| *p*-anisidine | 123.15 | 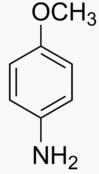 | veratryl alcohol | 168.19 | 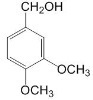 |

Table S5 Humic substances

| DOM | Sources | Abbreviation | C | H | O | N |
| --- | --- | --- | --- | --- | --- | --- |
| Fulvic acid (FA) | Suwannee river II | SRFA | 52.34 | 4.36 | 42.98 | 0.67 |
|  | Nordic lake | NLFA | 52.31 | 3.98 | 45.12 | 0.68 |
|  | Pony lake | PLFA | 52.47 | 5.39 | 31.38 | 6.51 |
| Humic acid (HA) | Suwannee river II | SRHA | 52.63 | 4.28 | 42.04 | 1.17 |
|  | Nordic lake | NLHA | 53.33 | 3.97 | 43.09 | 1.16 |
| Natural organic matter (NOM) | Suwannee river | SRNOM | 50.70 | 3.97 | 41.48 | 1.27 |

Table S6 The parameters of DOM constituents calculated at B3LYP/6-311G* level

| DOM constituents | E_HOMO_ | E_LUMO_ | q- | qH+ | μ | α | E | ZPE^a^ | H | G | Eth^a^ | Cv^b^ | S^b^ | V |
| --- | --- | --- | --- | --- | --- | --- | --- | --- | --- | --- | --- | --- | --- | --- |
| hydroquinone | -0.199 | -0.002 | -0.650 | 0.405 | 2.683 | 64.129 | -382.678 | 68.158 | -382.562 | -382.600 | 72.408 | 26.698 | 80.518 | 80.359 |
| catechol | -0.204 | 0.009 | -0.632 | 0.406 | 1.177 | 64.203 | -382.675 | 68.272 | -382.559 | -382.597 | 72.474 | 26.526 | 80.116 | 77.218 |
| resorcinol | -0.212 | 0.007 | -0.646 | 0.409 | 1.356 | 63.916 | -382.682 | 68.346 | -382.565 | -382.603 | 72.513 | 26.464 | 79.997 | 80.873 |
| aniline | -0.198 | 0.009 | -0.786 | 0.318 | 1.715 | 64.077 | -287.602 | 73.661 | -287.478 | -287.514 | 77.292 | 23.085 | 75.520 | 77.217 |
| syringic acid | -0.215 | -0.038 | -0.633 | 0.428 | 4.716 | 112.987 | -725.070 | 116.310 | -724.870 | -724.925 | 124.842 | 50.301 | 115.546 | 136.214 |
| gallic acid | -0.236 | -0.047 | -0.684 | 0.424 | 6.469 | 85.886 | -646.458 | 80.279 | -646.318 | -646.366 | 86.956 | 40.847 | 100.438 | 104.443 |
| *p*-anisidine | -0.182 | 0.007 | -0.786 | 0.314 | 1.813 | 81.738 | -402.121 | 94.172 | -401.962 | -402.004 | 99.444 | 32.250 | 88.923 | 97.315 |
| *o*-anisidine | -0.185 | 0.019 | -0.789 | 0.334 | 1.704 | 80.748 | -402.124 | 94.403 | -401.964 | -402.006 | 99.583 | 32.101 | 88.026 | 96.560 |
| 3,5-dihydroxybenzoic acid | -0.233 | -0.047 | -0.641 | 0.413 | 7.002 | 81.753 | -571.242 | 77.495 | -571.108 | -571.153 | 83.497 | 36.867 | 95.560 | 101.640 |
| guaiacol | -0.203 | 0.012 | -0.654 | 0.423 | 2.734 | 75.778 | -421.989 | 86.563 | -421.842 | -421.884 | 91.578 | 30.736 | 87.392 | 95.322 |
| *p*-methoxyphenol | -0.196 | 0.000 | -0.650 | 0.405 | 0.480 | 76.713 | -421.985 | 86.228 | -421.838 | -421.880 | 91.334 | 31.107 | 87.931 | 94.632 |
| 3,4-dimethoxyphenol | -0.199 | 0.009 | -0.649 | 0.405 | 3.280 | 92.449 | -536.501 | 106.637 | -536.319 | -536.367 | 113.454 | 40.360 | 101.263 | 111.545 |
| *p*-aminophenol | -0.183 | 0.005 | -0.786 | 0.403 | 2.116 | 68.949 | -362.815 | 76.079 | -362.685 | -362.724 | 80.515 | 27.884 | 81.647 | 83.323 |
| vanillin | -0.219 | -0.037 | -0.642 | 0.429 | 1.673 | 96.153 | -610.566 | 96.106 | -610.401 | -610.449 | 102.874 | 40.690 | 102.150 | 114.313 |
| syringaldehyde | -0.222 | -0.052 | -0.644 | 0.432 | 3.565 | 110.933 | -649.833 | 112.818 | -649.639 | -649.693 | 120.876 | 46.696 | 112.022 | 129.287 |
| *p*-hydroxybenzoic acid | -0.236 | -0.038 | -0.632 | 0.413 | 1.847 | 79.341 | -496.041 | 75.320 | -495.911 | -495.954 | 80.485 | 31.768 | 89.498 | 94.188 |
| salicylic acid | -0.250 | -0.047 | -0.685 | 0.441 | 6.520 | 76.078 | -496.034 | 75.184 | -495.905 | -495.947 | 80.375 | 31.837 | 89.744 | 94.038 |
| caffeic acid | -0.215 | -0.062 | -0.641 | 0.433 | 2.128 | 120.559 | -648.658 | 98.829 | -648.488 | -648.539 | 106.266 | 44.685 | 108.509 | 121.421 |
| vanillic acid | -0.219 | -0.037 | -0.642 | 0.429 | 1.673 | 96.153 | -610.566 | 96.106 | -610.401 | -610.449 | 102.874 | 40.690 | 102.150 | 114.313 |
| *p*-aminobenzoic acid | -0.236 | -0.038 | -0.632 | 0.412 | 3.257 | 79.350 | -496.040 | 75.323 | -495.911 | -495.954 | 80.488 | 31.769 | 89.495 | 96.184 |
| 2,5-dihydroxy-1,4-benzoquinone | -0.256 | -0.120 | -0.612 | 0.432 | 2.961 | 73.670 | -531.902 | 59.587 | -531.798 | -531.840 | 64.635 | 30.818 | 88.221 | 87.222 |
| veratryl alcohol | -0.220 | 0.004 | -0.615 | 0.395 | 1.579 | 103.256 | -575.804 | 124.421 | -575.592 | -575.645 | 132.309 | 45.208 | 111.146 | 123.779 |

^a^ Kcal/Mol, ^b^ Cal/Mol-kelvin

Reference

Clark, C.D., de Bruyn, W., Jones, J.G. Photoproduction of hydrogen peroxide in aqueous solution from model compounds for chromophoric dissolved organic matter (CDOM). Mar. Pollut. Bull. **79**, 54-60 (2014).
